# Distribution of fresh foods in food pantries: challenges and opportunities in Illinois during the COVID-19 pandemic

**DOI:** 10.1186/s12889-023-16215-4

**Published:** 2023-07-07

**Authors:** Jiayi Huang, Stephanie Acevedo, Mallory Bejster, Caitlin Kownacki, Dale Kehr, Jennifer McCaffrey, Cassandra J. Nguyen

**Affiliations:** 1 Office of Extension and Outreach, University of Illinois at Urbana-Champaign, 1301 W. Gregory Dr, Urbana, IL 61801 USA; 2 Department of Community, Systems and Mental Health Nursing, Rush University College of Nursing, 600 S. Paulina St, Chicago, IL 60612 USA; 3 Nutrition Department, University of Californa, Davis, One Shields Ave, Davis, CA 95616 USA

**Keywords:** Food insecurity, Food supply, Focus groups, Access to healthy foods, Food storage

## Abstract

**Background:**

The charitable food system distributes free food to clients across the U.S., but many nutrition and health-focused efforts encounter barriers to success, which were exacerbated during the COVID-19 pandemic. The objective of the current study was to understand barriers and facilitators to distributing nutritious, fresh foods in food pantries across Illinois during the COVID-19 pandemic.

**Methods:**

Forty-nine pantry representatives participated in focus groups in October 2021. A codebook was created based on relevant literature, stakeholder interests, and an initial review of the recordings. Transcripts of each group were coded and analyzed using a basic interpretive approach.

**Results:**

Pantries distribution of fresh foods was impacted by community partners, food bank policies and practices, and the quality of the donated fresh foods. Physical constraints of pantries limit fresh food storage capacity. The COVID-19 pandemic magnified stressors in the charitable food system which highlighted how community partners might improve fresh food distribution.

**Conclusion:**

Focus groups with food pantry representatives across Illinois provided key insights that can inform future efforts to facilitate fresh food distribution in the charitable food system. Future studies should evaluate the effects of the suggested initiatives and changes at the food pantry, food bank, and policy levels.

**Supplementary Information:**

The online version contains supplementary material available at 10.1186/s12889-023-16215-4.

## Summary box

What is already known on this topic? Barriers exist in effectively distributing fresh foods to clients of the charitable food system.

What is added by this report? The current study explored barriers and facilitators to distributing nutritious, fresh foods in pantries across Illinois during the COVID-19 pandemic, which highlighted existing and introduced new challenges.

What are the implications for public health practice? There are opportunities for change to improve fresh food distribution in the charitable food system across all socio-ecological levels, including in individual food pantries, among regional food banks, and at the national policy level.

## Background

In 2020, the year in which the World Health Organization declared COVID-19 a worldwide pandemic, over 38 million people in the U.S. experienced food insecurity, defined as uncertain access to adequate food [1]. The U.S. government has implemented several programs, such as the *Supplemental Nutrition Assistance Program*; the *Special Supplemental Nutrition Assistance Program for Women, Infants, and Children*; and the free or reduced price *National School Lunch Program* to address food insecurity. These programs provide supplementary monetary support for groceries, food vouchers, and prepared meals, respectively, to individuals who meet eligibility criteria. However, gaps in federal food security efforts leave some household food needs unmet. A private decentralized charitable food system has emerged to offset unmet needs from governmental programs.

The charitable food system is comprised of various actors. These include donors and non-profit organizations which supply food, financial, and in-kind support to community-serving agencies. These agencies include large ware-housing food banks (which source, transport, and store large quantities of food) and smaller food pantries (which distribute food directly to individuals).^1^ For many years, this system focused primarily on distributing as much food as possible, particularly shelf-stable goods [2].

Recently, advocates have championed a shift to distribute more nutritious foods while emphasizing client choice [3–5]. Food banks and pantries have implemented a variety of initiatives, including distributing lists of nutritious foods to donors, updating distribution practices from pre-packaged boxes to client-choice models, and partnering with external organizations [4, 6, 7]. In 2020, guidelines were published by Healthy Eating Research (a national program of the Robert Wood Johnson Foundation that funds research and policy efforts) to promote consistent nutrition classification of foods distributed across the system in the U.S. [5].

However, many pantry-based initiatives encounter barriers to providing nutritious foods in dignified settings. Challenges include storage limitations, perceived lack of interest among clients, constrained budgets, and reliance on donations [8–10]. Fresh foods (e.g., fruits, vegetables, dairy, lean meats), in particular, are highly perishable and require temperature-controlled conditions to maintain palatability, safety, and nutrient value. In addition to these barriers, the COVID-19 pandemic presented new challenges in distributing foods and maintaining choice for clients while adapting to shifting public health guidance [11, 12]. It has yet to be established how food pantries barriers and facilitators to nutrition promotion were different during the COVID-19 pandemic. The current study explored barriers and facilitators to distributing nutritious, fresh foods in pantries across Illinois during the COVID-19 pandemic. The results of this qualitative inquiry can be used to inform promising areas for intervention to improve the capacity of the charitable food system to attain, store, and distribute fresh foods.

## Methods

### Context

This study was an expansion of earlier research focused on facilitators and barriers to healthy food distribution among pantries in Lake County, IL (a predominantly suburban county comprised of 1,368 square miles in the northeastern corner of Illinois which borders Lake Michigan). The results informed opportunities for change in the local charitable food system, but the team was interested in understanding perspectives across the state. While preparing for statewide focus groups, team members learned of an effort to create a formal Farm to Food Bank system in Illinois. The research team connected with this larger effort, narrowing the focus of investigation to fresh foods, a specific type of nutritious food with a shorter shelf-life and unique transportation and storage challenges.

### Data collection

A script was developed to facilitate a discussion about challenges and opportunities faced in distributing fresh foods in pantries. The script (Supplemental Material) included a definition of what fresh foods were (and were not), followed by 11 open-ended questions regarding sourcing, pick-up, storage, distribution, and partnerships, with follow-up prompts and probing questions. These categories of influence were selected based on the literature documenting the shift of the charitable food system to distribute more nutritious foods while emphasizing client choice [3–5]. The script was reviewed by external colleagues with expertise in regional food systems and charitable foods networks who suggested edits that improved flow and clarity. Three individuals (authors SA and MB as well as an additional staff member) without prior qualitative research experience were trained as focus group moderators. Training included an overview of best practices and mock focus groups to practice moderation skills.

Participants were recruited with convenience sampling via e-mails sent from food banks and Extension staff. To be eligible, individuals had to be at least 18 years old, fluent in English, and work at a pantry. Three 2-h focus groups, at varying times and dates, were offered in north, central, and southern Illinois. Each of the 9 focus groups were hosted virtually using Zoom (Version 5; San Jose, CA). Focus group participants did not have prior professional relationships with the moderator or notetaker in the session. Before the focus group began, participants completed a brief descriptive quantitative questionnaire. This questionnaire included participant sociodemographic characteristics (Table 1), participants’ affiliated food pantries’ characteristics (Table 2), and participants’ beliefs about their food pantries and their roles (Table 3). All questionnaire items and possible responses are shown in Tables 1, 2 and 3. At each focus group the moderator read the consent form and participants verbally consented. The study protocols were approved as exempt from review and a waiver for written documentation of informed consent was provided by the University of Illinois at Urbana-Champaign Institutional Review Board (protocol #22162). All methods were carried out in accordance with relevant guidelines and regulations. Participants were compensated with a $20 gift card.

**Table 1:** Characteristics of focus group participants (*n* = 49) and their affiliated pantries

*Characteristic*	*Mean*	*SD*
Age, years	54.65	16.19
Income, monthly	6190.97	4960.47
Monthly volunteer/work at food pantry, hours	60.30	58.59
*Gender identity*	*%*	*n*
Man	16.3	7
Woman	81.4	35
Prefer not to answer	2.3	1
*Race (all that apply)*	*%*	*n*
American Indian or Alaska Native	0.0	0
Asian	0.0	0
Black or African American	25.6	11
Native Hawaiian or other Pacific Islander	0.0	0
White	72.1	31
Prefer not to answer	2.3	1
*Ethnicity*	*%*	*n*
Hispanic or Latinx	0.0	0
Non-Hispanic or Latinx	95.2	40
Prefer not to answer	4.8	2
*Highest level of education*	*%*	*n*
Less than high school	0.0	0
High school/GED	9.3	4
Some college/Associate's degree	20.9	9
Bachelor's degree	27.9	12
Professional degree	7.0	3
Graduate/Post-Graduate degree	34.9	15
*Year(s) at food pantry*	*%*	*n*
Less than 1 year	10.6	5
1–3 years	21.3	10
4–5 years	12.8	6
6–10 years	19.2	9
11 + years	36.2	17
*Role at food pantry*	*%*	*n*
Volunteer	18.8	9
Paid staff	20.8	10
Board member	2.1	1
Pantry manager/director	62.5	30
Other (e.g., Coordinator and Regional Director)	8.3	4
*Year(s) in role at food pantry*	*%*	*n*
Less than 1 year	20.8	10
1–3 years	22.9	11
4–5 years	12.5	6
6–10 years	16.7	8
11 + years	27.1	13

Missing responses for age (*n* = 6), income (*n* = 20), monthly hours (*n* = 2), gender (*n* = 6), ethnicity (*n* = 7), education (*n* = 6), years at food pantry (*n* = 2), role (*n* = 1), and years in role (*n* = 1)

**Table 2:** Characteristics of Food Pantries Affiliated with Focus Group Participants (*n* = 49)

*Characteristic*	*Mean*	*SD*
Monthly families served	334.73	569.00
Monthly individuals served	883.34	2005.53
*Sources (%) of fresh food*	*Mean*	*SD*
Food bank	55.67	37.40
Food retail (Stores)	19.60	28.47
Private donors	7.47	13.73
Farms or farmers	4.23	8.43
Community gardens	1.49	3.13
On-site garden	0.11	0.76
Other food pantries	2.12	10.70
Meat processors/meat donors	1.05	5.52
Other (e.g., Catholic Charities, wholesale, or only distributing non-perishable foods)	8.26	25.77
*Food pantry's affiliation (all that apply)*	*%*	*n*
Faith-based	60.4	29
Social or public health services	14.6	7
Hospital, clinical, or medical services	4.2	2
School	0.0	0
College or University	0.0	0
Mobile distribution site	2.1	1
Governmental	6.3	3
Other (e.g., outreach, community action, and food bank)	8.3	4
No affiliation/standalone operation	12.5	6
*Choice available to clients at food pantry*	*%*	*n*
None (they do not choose any items)	22.9	11
Some (they choose some items)	37.5	18
All (they choose all items)	39.6	19
*Food pantry location*	*%*	*n*
Farm/Rural community	10.4	5
Town under 10,000 people and rural, non-farm	6.3	3
Towns & cities with 10,000–50,000 people	45.8	22
Suburbs of city with over 50,000 people	18.8	9
Urban city with over 50,000 people	18.8	9
*Prior partnership with Illinois SNAP-ED*	*%*	*n*
Yes	60.0	21
No	40.0	14
*Prior completion of a NEFPAT*	*%*	*n*
Yes	20.0	7
No	80.0	28
*Food pantry openings*	*%*	*n*
Every day	20.8	10
1 day per week	41.7	20
2 or more days per week	25.0	12
3 or less days per month	12.5	6
*Affiliation with a food bank*	*%*	*n*
Yes	85.4	41
No	12.5	6
Don't know	2.1	1
*Refusal of fresh food donations*	*%*	*n*
Weekly	2.4	1
About once a month	11.9	5
More than once a month but less than every week	0.0	0
About once every three months	16.7	7
About once a year	0.0	0
Never	29.1	29
*Interest in receiving processed venison from hunters*	*%*	*n*
Yes	30.2	13
No	23.3	10
Maybe	46.5	20
*How food pantry markets its services (all that apply)*	*%*	*n*
Word of mouth	90.7	39
Radio ads	0.0	0
Billboards	14.0	6
Food pantry website	51.2	22
Social media accounts	83.7	36
Other (e.g., flyers, school district, and nonprofits)	34.9	15
We do not market our services	2.3	1

*NEFPAT* Nutrition Environment Food Pantry Assessment Tool, *SNAP-ED* Supplemental Nutrition Assistance Program-Education Component. Missing responses for families served (*n* = 12), individuals served (*n* = 11), sources (*n* = 6), affiliation (*n* = 1), choice (*n* = 1), location (*n* = 1), partnership (*n* = 14), NEFPAT (*n* = 14), openings (*n* = 1), food bank affiliation (*n* = 1), refusal (*n* = 7), venison (*n* = 6), and marketing (*n* = 6)

**Table 3:** Frequency of agreement to statements about respondents’ (*n* = 49) food pantries and roles

*Statement*	*Agree*	*Somewhat agree*	*Somewhat disagree*	*Disagree*
The foods offered at my food pantry meet the health needs of clients	37.2% (16)	51.2% (22)	9.3% (4)	2.3% (1)
My food pantry offers a wide variety of fresh food items	38.1% (16)	42.9% (18)	11.9% (5)	7.1% (3)
My food pantry consistently has fresh fruits and vegetables available	34.9% (15)	39.5% (17)	14.0% (6)	11.6% (5)
It is difficult to stock enough fresh food in my food pantry	37.2% (16)	34.9% (15)	14.0% (6)	14.0% (6)
I have influence over the amount of fresh food available in my food pantry	40.5% (17)	21.4% (9)	21.4% (9)	16.7% (7)
I can take actions to increase the amount of fresh food in my food pantry	37.2% (16)	32.6% (14)	18.6% (8)	11.6% (5)
My food pantry can respond to the dietary needs of cultural and ethnic groups we serve	26.2% (11)	35.7% (15)	26.2% (11)	11.9% (5)

Estimates shown are % (n). Missing responses for the first (*n* = 6), second (*n* = 7), third (*n* = 6), fourth (*n* = 6), fifth (*n* = 7), sixth (*n* = 6), and seventh (*n* = 7) statements

### Analysis

**Fig. 1:**
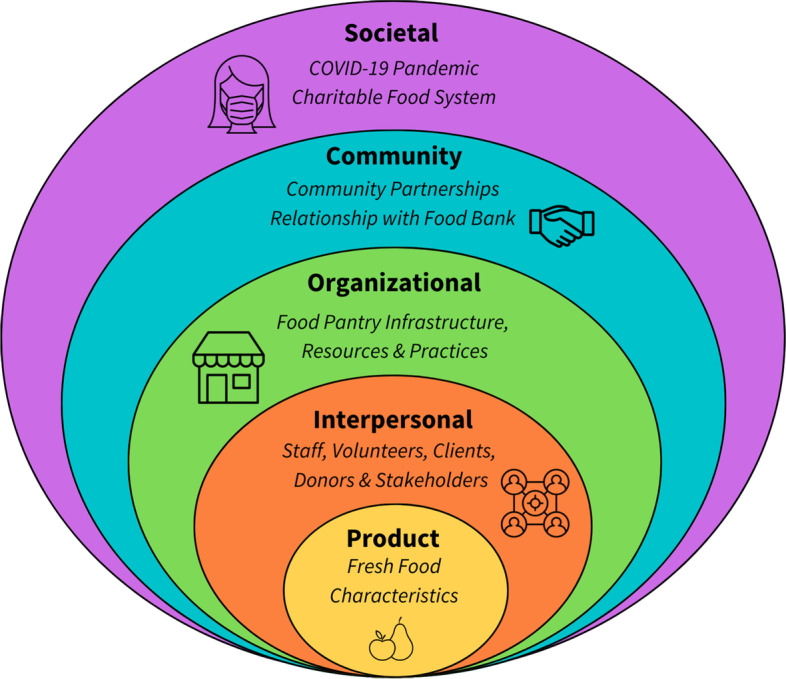
Model of influences on food pantry fresh food distribution

Focus groups were digitally recorded, and the files were transcribed verbatim. One research team member reviewed the transcripts while listening to the recording to ensure accuracy and blind any personal identifiers. All data in the study were anonymized before use. A codebook was drafted based on related literature, stakeholders’ key interests, and an initial reading of transcripts. This draft was collaboratively refined to reflect 30 distinct codes (categorized into 21 challenges and 9 opportunities). Five members of the research team tested this codebook on the same transcript, then clarified code definitions and added exclusion criteria and/or quotes, as necessary. Each transcript was independently coded by 2 team members using the final codebook, and team members discussed any discrepancies to come to consensus. One team member served as a mediator to make a final decision if consensus was not reached. After all transcripts were coded, the codes were condensed into five distinct levels of influence, mirroring aspects of the Social-Ecological Model (SEM) [13] (Fig. 1). Excerpts within these levels were analyzed using a basic interpretative approach, with common sentiments, key ideas, and variety of experiences reflected using summaries and exemplary quotes. Questionnaire responses were characterized with descriptive statistics.

## Results

Forty-nine individuals participated in nine focus groups hosted in October 2021. Descriptive characteristics of participants based on survey questionnaire responses are shown in Table 1. The pantries represented were diverse, reflected in the wide range of individuals served each month, varying affiliations, choice for clients, and rurality of location, among other reported characteristics (Table 2). Most pantries (85%) were affiliated with a food bank in their respective region, though seven representatives reported no or were unaware of an affiliation. When asked about their level of agreement regarding their pantries on the quantitative questionnaire, many respondents agreed that they offered a wide variety of fresh foods and that the foods met the client health needs (Table 3). However, a smaller number of respondents felt they could take actions to increase the quantity of fresh foods in their pantry. The statement least likely agreed to was “*my food pantry can respond to the dietary needs of cultural and ethnic groups we serve,*” confirmed by 62% of respondents.

The analysis of focus group transcripts resulted in challenges and opportunities which were organized into five distinct levels of influence (Fig. 1). These included 1) societal, 2) community, 3) organizational, 4) interpersonal, and 5) product, described in the sections below.

### Societal

The COVID-19 pandemic was a significant force, impacting operations and pantries’ abilities to acquire, store, and distribute fresh foods. Many pantries moved from face-to-face shopping-style distribution models to shopping lists or pre-packaged boxes. This shift required more volunteers to pack and distribute boxes, coinciding with lower volunteer turnout. Pre-packaged boxes limited client choice, making distribution of fresh foods more challenging because products pre-selected for clients would not necessarily align with clients’ preferences. As one representative noted: *“Unfortunately, when we were packing the boxes during the pandemic, we did find some foods [left] like outside on the street.”*

Though pantries regularly experience shifts in the number of clients served each week, representatives noted greater shifts during the COVID-19 pandemic, with most indicating reduced numbers, paired with an increase in food donations, presenting difficulties in distributing food before spoiling.

All representatives identified fresh foods as a priority. However, there were aspects of working within the charitable food system which impacted their capacity to address this priority. Grants or other resources available to nonprofit organizations were instrumental in allowing pantries to directly purchase fresh foods or cold storage units. However, fresh food distribution was not their only priority. Many representatives stated that if they had extra funds, they would purchase supplies to help clients shop (i.e., shopping carts) or to meet non-food needs of clients (i.e., personal hygiene products).

### Community

Many pantries identified the broader community as integral in distributing fresh foods. Pantries had relationships with diverse organizations, including senior centers, law enforcement, schools, other pantries, farmers and farmers markets, businesses, park districts, faith groups, emergency shelters, and gardeners. Partnerships were leveraged to acquire foods, raise funds, connect with individuals in need, and distribute excess perishable foods. These partnerships were particularly critical in the face of changing conditions during the COVID-19 pandemic:

*“When we started getting more food, we began to share that … with shelters at the end of the week, but that has been continuous now, so we... Even if we have vegetables that we don't think will make it the next week, we'll call that agency and they will come and pick up those items to make sure that they are utilized.”*

Pantries affiliated with food banks spoke about the value of this relationship. Many received all or most of their fresh foods, at no or low cost from the food bank. Despite the value of these relationships, participants described difficulties and confusion with the size, quantity, and shelf-life of items ordered which disrupted plans and capacity for fresh food distribution. This ambiguity along with concerns about quality caused some pantries to avoid certain products:

“*The food bank [does] not put expiration dates in the order form for the dairy items. So, I don't know when I'm ordering, am I ordering one day expired yogurt, or nine days expired yogurt or not expired yogurt? So, then I hesitate to order the dairy products, even when they're free*.”

Representatives expressed challenges in meeting some food safety laws and guidance such as requirements to use temperature-controlled trucks, which pantries had limited access to. A few representatives noted how new food bank agreements restricted their ability to source fresh foods from local grocery retailers: “*unbeknownst to us, [food bank] went to some of the bigger places like [grocery store] … and had them sign a corporate contract that they would only give [food] to them*.” Participants also frequently mentioned that restrictions on redistributing excess food to other pantries were a barrier, resulting in wasted food. This restriction was particularly cumbersome in the face of large donations of fresh foods coupled with lower numbers of clients and volunteers during the COVID-19 pandemic.

### Organizational

The physical space of pantries, including structure and size, impacted pantries’ abilities to receive, store, and distribute fresh foods. Many participants noted the small size and limited storage of their pantry. Notably, limited cold storage was the most frequent challenge mentioned. This impacted the types and amounts of fresh foods they could store, as many fresh foods (e.g., fresh meat, and dairy products) need to be refrigerated or frozen. One participant shared “*we are always limited by our cold storage capability. The [food bank] might make for example, 15 crates of eggs available, but we can only handle five.*” Unsurprisingly, when participants were asked how they would spend a $5,000 grant, many said they would purchase refrigerators or freezers. However, some participants noted that they could not add more cold storage even if available due to facility space limitations.

Transportation was another challenge for many pantries. Many pantries did not have adequate vehicles or volunteers to pick up fresh foods. One representative reported partnering with other pantries to coordinate fresh food pick-ups for multiple pantries at one time. Another challenge was the timing of pick-up or deliveries in relation to the pantries’ food distribution. If a pick-up or delivery date is too far out from the day of distribution, fresh foods may mature beyond peak freshness and become waste. Many representatives described how an ideal system would allow for more frequent deliveries of fresh foods directly from local sources, bypassing storage at local food banks when possible.

While many pantries worked with multiple entities in the community to obtain fresh foods, few pantries had food procurement or nutrition-related policies. One exception was a policy focused on the quality of fresh food a pantry will accept: “*we have developed a nutrition policy in order to work with some of our rescue partners and let them know, we're not here to take your garbage*.”

### Interpersonal

The capacity of pantries to distribute fresh foods relied heavily on the people within the charitable food system. Many representatives emphasized their pantries were volunteer organizations limited by the number of volunteers as well as volunteers’ prior experience with pantry policies, nutrition knowledge, and familiarity with food safety guidelines. When pantries receive an abundance of fresh produce, removing overripe or unsafe items relied on volunteers’ relevant knowledge of food safety and pantry policies. This required more volunteer time and attention compared to assessing expiration dates on shelf-stable donations.

Donor relationships were critical to every pantry who participated. Many pantries shared hesitation in voicing their needs with donors for fear they would stop donating. However, one participant stated that when they communicated with donors, they responded positively. *"So, I had to tell them...I could only take either every other week or half the amount every week. And they said, "okay, we'll find somebody else for the other half".*

Representatives wanted clients to be considered when distributing fresh foods. Donors and volunteers may have dissimilar cultural or social backgrounds than clientele, causing disparity between foods donated and clients' needs. One example shared was: *"We like having the milk as well, but since we're outside, a lot of our people are walkers. In the hot weather, it's hard for them to get that home in time that it won't spoil quickly."* Another representative noted that some clients had dental challenges, which made crisp fresh foods (i.e., apples, celery) less appealing. Fresh foods can also be unappealing to clientele if they have limited experience preparing them or if preparation equipment is not readily available (e.g., clients living in temporary shelters or hotels). Finally, representatives believed that inconsistent guidance on the best buy, use by, sell by, and expired by dates left clients confused regarding food safety.

Representatives noted varying experiences with external stakeholders in their efforts to distribute more fresh foods. Fresh foods were received from a variety of sources, including food rescue organizations, farmers, local donors, and university-based gardens, as examples. One facilitator of fresh food distribution was a food voucher program, which allowed clients to redeem vouchers for fresh foods at a local grocery, bypassing pantry storage limitations. Many representatives also partnered with other pantries but were cautious in naming who and how they partnered, as their food bank was unsupportive: "*Our partners are pantries that are nearby. They're not competitors. We're all in the same business. … a lot of that stuff happens frankly, under the radar or out the back door."* Representatives frequently commented that they wished they worked more closely with other pantries to share food, resources, and information.

### Product

Several challenges experienced by pantries were related to fresh food characteristics. When fresh foods were past their peak, there was limited time to distribute, resulting in wasted food. Pantries desired fresher, higher quality food to maximize the distribution time period: “*It needs to be good quality stuff that we all as food pantries have the opportunity to have some time to distribute it before it goes bad*.” Food waste also stemmed from food quantity challenges. Many representatives noted they received a lot of certain foods which were unpopular with clients or not feasible to distribute completely given the amount received, distribution hours, and number of clients served.

Pantries also indicated they desired more food variety. Pantries often received a large amount of a few items rather than a diverse spread: “*We'll get massive amounts of one thing, like we'll have 10 cases of apples or something, and nothing else*.” Finally, some representatives raised concerns over a lack of culturally relevant foods for the population they served.

## Discussion

As the charitable food system continues to evolve from delivering mainly pre-packaged shelf-stable goods to more fresh foods, barriers exist in effectively distributing it to clients. The present study illustrated specific barriers and facilitators Illinois pantries faced in distributing fresh foods. Important factors included community partners, food bank practices, federal policies, and characteristics of donated fresh foods. Physical limitations presented challenges, with cold storage the most frequently noted barrier. The COVID-19 pandemic, during which this study was conducted, magnified stressors in the charitable food system that further highlighted how community partners can improve opportunities to distribute fresh foods.

Pantries have partnerships with various external stakeholders who provide food, volunteers, and resources. Such connections are essential for pantry operations. Many study participants indicated they wanted more connections but lacked the time, energy, or knowledge. Community organizations, like Cooperative Extension (a system of community-based professionals affiliated with state- and territory-based universities which work with local citizens and groups to solve problems using research-based knowledge), can help forge these partnerships given their knowledge of the communities they serve. One tool available for this work is the Nutrition Environment Food Pantry Assessment Tool (NEFPAT), which quantifies a food pantry’s use of policy, systems, and environmental strategies to promote nutrition and dignity among food pantry clientele [7]. Two recent studies using the NEFPAT demonstrated how Extension staff helped pantries develop new partnerships, adopt nutrition policies, and encourage healthy choices [6, 14].

Pantries' efforts to distribute fresh foods can be impacted by their respective food banks’ policies. Pantries receive most of their food from food banks [15]. Present results indicated that pantries were limited by the foods available, information provided about the food, transportation options, and timing of orders in relation to their food distribution. A recent study found that 24 pantries ordered healthier foods when the food bank ordering system incorporated nutritional rankings for items [16]. Providing additional information about expiration dates; packaging; and food quality, as suggested by study participants, may impact pantries’ orders of fresh foods.

Food banks also have a role in influencing practices in their network, such as how pantries partner. Partnerships among pantries can provide an opportunity to share best practices and mitigate potential food waste [17]. Although valued by study participants, some food banks restrict food sharing among pantries to maintain fair share allotments in a region, reduce food safety risks, and abide by USDA regulations. For example, The Emergency Food Assistance Program (TEFAP) foods can only be supplied to certain agencies that provide exclusive assistance to defined populations. Approval processes are required before any food can be transferred between agencies [18]. As illustrated by the model produced in the study, pantries’ fresh food distribution is impacted by the broader food system, policy landscape, and socio-historical conditions [13]. Thus, national policy change should supplement activities within individual pantries and food banks. As one example, policymakers might consider changing TEFAP restrictions so that pantries can work collaboratively to respond to their region’s shifting food supply without concerns of reprimand.

Food waste was a common problem cited by participants. Such waste results from low quality and excess quantity of foods. Some representatives are hesitant to reject food from donors, fearing damaged relationships that lead to decreased donations. These sentiments are illustrative of a scarcity mindset that has been described as a barrier to the evolution of the charitable food system [4]. To combat this issue, some pantries have adopted nutrition policies that outline quality requirements of donated food. These policies can communicate to stakeholders a dedication to quality for clients, decrease time spent sorting foods, and, ultimately, decrease food waste.

Representatives shared a common desire for direct donations from local growers. This would bypass storage in grocery stores or food banks, lengthening the shelf life of fresh foods in pantries. State-level policies that encourage diversion of fresh foods directly to pantries may decrease food waste. Policies like this were notably absent in a recent review of state-level food donation policies across the U.S. [19]. Instead, liability protection for donors was the most common. Sixteen states have tax incentive policies for donations [19], but such policies do not consider the quality of food donated. Florida was the only state with a policy regarding food recovery to reduce waste but specifically targeted surplus fruits [19]. Future opportunities include state-level policies that earmark fresh foods for pantries or revised tax incentives to consider quality, nutrition, or freshness of foods.

Pantries’ facility capacities hinder their fresh food distribution. Challenges of cold storage and refrigerated transportation were echoed in every focus group. The vital nature of cold storage has been reported previously [20, 21]. Cold storage increases pantries’ capacity to store foods in high demand, such as meat, eggs, and dairy [22, 23]. While some communities have grants available to purchase cold storage for pantries [24], many in the current study noted they lacked space or wiring for additional cold storage units. Another solution that bypasses facility and transportation limitations is voucher programs that allow clients to exchange vouchers for fresh foods at grocery stores. However, this program relies on partnering grocery stores, and client transportation to grocery stores can be challenging [21]. Thus, pantry and community characteristics should be considered when addressing barriers to fresh food distribution.

The precarious balance between foods donated and client needs in the charitable food system was magnified during the COVID-19 pandemic. In March 2020, pantries experienced increased demand due to businesses closing and increases in unemployment [25]. As the pandemic continued, policies to increase SNAP benefits and participation in other nutrition programs may have reduced pantry demand [26]. The fluctuation in clientele at pantries coincided with the USDA Farmers to Families Food Box Program [27], which provided pantries an abundance of fresh produce, dairy, and meat. Clients who received excess food may decrease the frequency of their pantry visits, which could explain the lower client numbers reported by study participants. Pantries in the current study, like other pantries across the U.S. [25], transitioned from face-to-face shopping-style distributions to pre-packaged boxes or drive-thru distributions to mitigate the COVID-19 transmission risk. These transitions, along with increased food donations, necessitated additional volunteers. Yet, representatives noted difficulties recruiting volunteers due to high risk or fear of exposure to the COVID-19 virus [28]. In facing these challenges, the resiliency of pantries was illustrated as representatives described how existing partnerships with pantries and stakeholders helped them meet the needs of community members. The value of resiliency afforded by community partnerships has been noted previously [29], and will likely be important in the face of future emergency scenarios.

Although this study adds newfound insight into pantries’ barriers and facilitators to fresh food distribution, it should be interpreted with its limitations. The sample size was limited, and participants were recruited using convenience sampling. However, to improve generalizability, pantries across Illinois were recruited to increase geographic and sociocultural variability. As participants had to be fluent in English, insights from individuals fluent solely in other languages were missed. However, though Spanish and Polish are common non-English languages spoken in Illinois, it is unclear whether any pantries in Illinois rely solely on staff and volunteers who are not fluent in English. Finally, the surveys completed were not directly linked with qualitative transcripts, so it was not possible to compare focus group responses by characteristics captured on questionnaires, such as pantry size. This was outside the scope of the current study but would be a valuable line of further inquiry.

## Conclusions

Focus groups with pantry representatives across Illinois provided key insights that can inform future efforts to facilitate fresh food distribution in the charitable food system. Opportunities for change were identified across all levels of the SEM. At the pantry level, suggested donation lists and quality standards can be developed and shared with donors to minimize volunteers’ sorting time and resulting food waste. At the food bank level, additional information in the ordering system that reflects important fresh food characteristics, when available, would be valuable. Lastly, changes to food donation policies at the state or federal levels may further improve quality and reduce waste. Future studies evaluating the effects these suggested initiatives have on the quality and quantity of fresh foods distributed are warranted.

## Supplementary Information

**Additional file 1.**

## Data Availability

The datasets generated and/or analyzed during the current study are not publicly available due to disclosure risk concerns but are available from the corresponding author on reasonable request.

## Abbreviations

NEFPAT: Nutrition Environment Food Pantry Assessment Tool
SEM: Social-Ecological Model

## Footnotes

1 Food pantries are referred to simply as “pantries” throughout the remainder of the paper.

## References

[1] Coleman-Jensen A, Rabbitt MP, Gregory CA, Singh A. Household Food Security in the United States in 20202021 9/7/2022. Available from: https://www.ers.usda.gov/publications/pub-details/?pubid=102075.

[2] Poppendieck J (1994). Dilemmas of emergency food: a guide for the perplexed. Agric Hum Values.

[3] Campbell E, Webb K, Ross M, Crawford P, Hudson H, Hecht K (2015). Nutrition-focused food banking.

[4] Martin KS (2021). Reinventing Food Banks and Pantries: New Tools to End Hunger.

[5] Schwartz M, Levi R, Lott M, Arm K, Seligman H (2020). Healthy Eating Research Nutrition Guidelines for the Charitable Food System.

[6] Nikolaus CJ, Kownacki C, Darvesh Z, McCaffrey J (2021). Technical assistance is related to improvements in the food pantry consumer nutrition environment. J Nutr Educ Behav.

[7] Nikolaus CJ, Laurent E, Loehmer E, An R, Khan N, McCaffrey J (2018). Nutrition Environment Food Pantry Assessment Tool (NEFPAT): Development and Evaluation. J Nutr Educ Behav..

[8] Chapnick M, Barnidge E, Sawicki M, Elliott M (2017). Healthy Options in Food Pantries—A Qualitative Analysis of Factors Affecting the Provision of Healthy Food Items in St. Louis, Missouri. J Hunger Environ Nutr.

[9] Verpy H, Smith C, Reicks M (2003). Attitudes and Behaviors of Food Donors and Perceived Needs and Wants of Food Shelf Clients. J Nutr Educ Behav.

[10] Kicinski LR (2012). Characteristics of short and long-term food pantry users. Michigan Sociol Assoc.

[11] Lanier J, Schumacher J. The Action of Foodbanks and Food Pantries in Central Illinois during the COVID-19 Pandemic. J Hunger Environ Nutr. 2021:1–9. Ahead of print.

[12] Larison L, Shanks CB, Webber E, Routh B, Ahmed S. The influence of the COVID-19 pandemic on the food supply in the emergency food system: a case study at two food pantries. Curr Dev Nutr. 2021;5(10):nzab115. 10.1093/cdn/nzab115 PMC8500014 34651097

[13] Brofenbrenner U, Friedman SL, Wachs TD (1999). Measuring environment across the life span: Emerging methods and concepts.

[14] Gibson S, Metcalfe JJ, McCaffrey J, Allison T, Prescott MP (2022). Nutrition environment at food pantries improves after fresh produce donation program. J Nutr Educ Behav.

[15] Weinfield NS, Mills G, Borger C, Gearing M, Macaluso T, Montaquila J, et al. Hunger in America 2014. Feeding America; 2014.

[16] Martin K, Xu R, Schwartz MB. Food pantries select healthier foods after nutrition information is available on their food bank's ordering platform. Public Health Nutr. 2020;24(15):1–8. 10.1017/S1368980020004814 PMC11082800 33243307

[17] Price CE, Sampson NR, Reppond HA, Thomas-Brown K, Camp JK (2019). Creating a community of practice among college campus food pantry directors in Michigan. J Community Pract.

[18] Donation of Foods for use in the United States, its Territories and Possessions and Areas Under its Jurisdiction. Sect. Part 250. 1988.

[19] Hudak KM, Friedman E, Johnson J, Benjamin-Neelon SE. US state variations in food bank donation policy and implications for nutrition. Prev Med Rep. 2022;27:101737. 10.1016/j.pmedr.2022.101737 PMC8958359 35355802

[20] Campbell EC, Ross M, Webb KL (2013). Improving the nutritional quality of emergency food: a study of food bank organizational culture, capacity, and practices. J Hung Environ Nutr.

[21] Yan S, Caspi C, Trude ACB, Gunen B, Gittelsohn J. How Urban Food Pantries are Stocked and Food Is Distributed: Food Pantry Manager Perspectives from Baltimore. J Hung Environ Nutr. 2020;15(4):1–13.

[22] Bazerghi C, McKay FH, Dunn M (2016). The role of food banks in addressing food insecurity: a systematic review. J Community Health.

[23] Remley D, Franzen-Castle L, McCormack L, Eicher-Miller HA (2019). Chronic health condition influences on client perceptions of limited or non-choice food pantries in low-income. Rural Communities Am J Health Behav.

[24] Grants allow Southern Illinois food pantries to increase cold food storage [press release]. Carbondale, IL: Illinois Extension, March 26, 2021 2021.

[25] McFetridge S. Food banks face virus dilemma: More demand, fewer volunteers2020. Available from: https://apnews.com/article/virus-outbreak-iowa-us-news-des-moines-ia-state-wire-6f0ea5e3ced0bb159eeeea51b16d8342.

[26] Toossi S, Jones JW, Hodges L. Pandemic-Related Program Changes Continued to Shape the U.S. Food and Nutrition Assistance Landscape in Fiscal Year 2021. Amber Waves. 2022. Available from: https://www.ers.usda.gov/amber-waves/2022/september/pandemic-related-program-changes-continued-to-shape-the-u-s-food-and-nutrition-assistance-landscape-in-fiscal-year-2021/.

[27] United States Department of Agriculture. USDA Farmers to Families Food Box 2021. Available from: https://www.ams.usda.gov/selling-food-to-usda/farmers-to-families-food-box. [updated May 28, 2021].

[28] Khalil A. Food Banks scrambling for volunteers amid omicron wave of covid-19 cases. Chicago Tribune. 2022. Available from: https://www.chicagotribune.com/coronavirus/ct-aud-nw-omicron-covid-food-banks-20220122-7puyl3nxbfhm5c5jtz5mperbmu-story.html.

[29] Thompson K, Sugg M, Barth M. The North Carolina Food Pantry Organizational Capability and Mapping Study: Research Brief and Pilot Study. J Agric Food Syst Commun Dev. 2019;9(1):1–13.

