# Supplementary material for: Distribution of fresh foods in food pantries: challenges and opportunities in Illinois during the COVID-19 pandemic
Source: BMC Public Health. 2023 Jul 7;23:1307. doi: 10.1186/s12889-023-16215-4 (PMC10327385; doi:10.1186/s12889-023-16215-4)
Supplement: Supplementary file 1 — Additional file 1. [file 12889_2023_16215_MOESM1_ESM.pdf]

Food Pantry Focus Group Script

Introduction:

Hello, thank you for joining us for this [focus group/interview]. We appreciate your participation. My name is __[NAME]__ and I will be helping to facilitate this discussion. __[NAME]__ is with me today to help take notes for the group. We are holding this discussion to learn about the challenges and opportunities of food pantries to get and distribute fresh foods.

Before we get started, we have a couple of housekeeping items to take care of. Yesterday you should have received a copy of the research consent form in your email inbox. Let's take a moment to review the consent form together. *[Moderator - Read consent form].* If anyone has questions about the consent I'm happy to answer those now. As a reminder, your participation in this project is voluntary. You can skip any questions you do not want to answer and you are also welcome to leave at any point. Before we move on I need to verify that everyone consents to participate. When I ask you, please respond with "yes" if you consent to participating in this project. If you decide not to consent respond "no" and our note-taker will remove you from the session.

[Moderator asks each person if they consent to participate. Note-taker documents each participants' response.]

Ok, now that we've gotten that taken care of we have a brief, 10-minute survey that we are asking you to complete, as it will help us collect complimentary information that we cannot easily gather during our conversation today. ___[NAME-notetaker]__ is placing a link to the survey in the chat box. Everyone can use the same link. The survey is voluntary and anonymous to complete.

(Survey link)

To make sure we are all thinking about the same thing when we refer to fresh foods, we are specifically talking about fresh fruits and vegetables, eggs, dairy (including milk, cheese, yogurt), butter, dry beans, and unprocessed meats. We have prepared an informational sheet with examples for your convenience.

Provide Fresh Foods handout

(*give participants 2-3 minutes to review handout*)

And, when we are discussing getting fresh foods we are referring to foods that are both donated from individuals or communities as well as purchased from stores, food banks, and other sources.

You were invited to join this discussion because you are an active member of an Illinois food pantry. We thank you for coming today to share your ideas with us and each other.

The information you share today is confidential. We ask that as a member of this discussion group you do not share personal information shared by others outside of this session. ___[NAME]___ and I also will respect the confidentiality of our conversation and ask that you do the same: what is said in this session stays in this session. No names or personally identifying information will be shared by us outside of this forum, we will only share general findings from our discussion. The information gathered in this discussion may be included in peer-reviewed publications and a stakeholder report with the goal of improving fresh food distribution in the state of Illinois.

Your participation in this discussion is very important to the research team. We would like to know more about challenges and opportunities that pantries in [REGION] face. Our discussion today will be informal. Our questions are about your individual experiences with food pantries, your ideas and opinions. There are no right or wrong answers. We simply want to know what you think and why.

I will help guide our conversation, but I encourage each of you to ask questions yourselves. It is important that a wide range of ideas are expressed. If you would like to add to an idea, or if you have an idea that is different from others, please speak up. I do ask that you try to respect another person's opinion or experience, even if you don't agree with it.

We are going to record this session so that we accurately capture your thoughts. We will use our notes and the recording to help us identify opportunities and challenges to guide efforts of policy and program development, as well as system level changes. But, your responses will remain confidential. While the research team will ensure all data is confidential, we cannot guarantee that others in the focus group will not divulge what was said outside the research setting. If you'd prefer not to use your real name than you're welcome to use a pseudo-name. No one will have access to the recording except us. Because we are recording, I may need to remind you occasionally to speak up or talk one at a time so that we can hear you clearly when we listen to the recording.

Thank you again for agreeing to participate in today's discussion. Our conversation will last about _[TIME]_. We will not be taking a formal break during this time, but please

feel free to get up and move around as you need. Please turn your cell phones off or on vibrate so that our discussion is not disturbed. Are there any questions before we begin? Alright, before we talk specifically about challenges and opportunities, I'd like to start with a brief introductory question for everyone.

#### START RECORDING NOW ####

8 m Icebreaker Question:

1. In 2-3 sentences, can you briefly share which food pantry you're affiliated with and what you enjoy most about working or volunteering there?

Key Questions

Sourcing: Let's begin by talking about how your food pantry gets fresh foods. This could come from a variety of different sources or people such as the food bank, farmers, other pantries, gardens, stores, or other sources.

12 m

2. How would you describe your pantry's strategy for getting fresh foods?
   a. **Prompt**: In other words, how do you get fresh foods?
   b. **Probe:** Is there anything that makes it easier for the pantry to get fresh foods?
   c. **Probe:** Are there any barriers or challenges that you face in getting fresh foods?
   d. **Probe**: How are decisions to get fresh foods made?

18 m

3. Can you describe a time when your pantry refused donations of produce or other fresh foods?
   a. **Probe**: If so, from whom and why?
   b. **Probe**: In that situation, what do you suggest donors do with these donations?

24 m

4. What type of fresh foods do you wish you received more often or in greater quantities and why?
   a. **Probe**: If you were offered more of that fresh food, can you describe any changes your food pantry would need to make to be able to accept and/or distribute this food?

30 m

5. If you could design an ideal system for getting as much fresh food as your pantry wanted, what would it look like?
   a. **Prompt**: Tell us about what this ideal process would look like for your pantry. This might include the people or places you get the food from, communication strategies, ordering systems, pick-up/transportation of foods, etc.
   b. **Probe**: How would this be different from your current strategy?

c. **Probe:** What prevents your food pantry from achieving this ideal system?

Pick-up, Storage & Distribution. We will now move to talking more about how your food pantry stores and distributes fresh foods.

36 m 6. Can you describe what helps your food pantry store and distribute fresh foods?
   a. **Prompt**: This could include pantry layout, space, budget, marketing, cold storage, refrigerated vehicle, or other things.

42 m 7. What are the top 3 barriers to your pantry being able to store and distribute more fresh food?

Partnerships: Finally, let's talk about ways you partner with other people, groups, or organizations to get, store, and distribute fresh foods.

48 m 8. Describe any times you've partnered with other food pantries to get, store, and/or distribute fresh foods?
   a. If so, is that partnership ongoing? If not, why not?
   b. If so and the partnership is ongoing, how did that partnership develop?
   c. In these partnerships, how do pantries divide or distribute fresh foods?
   d. If not, can you explain why you would or would not be interested in exploring such a partnership?

54 m 9. What community resources or organizations help your food pantry to get, store, and/or distribute fresh foods?
   a. **Prompt**: Resources could be financial, physical, or personnel. Organizations could include churches, public health departments, schools, and other local establishments or businesses.
   b. **Probe**: If nothing comes to mind, what resources or organizations might help your food pantry provide fresh foods?

60 m 10. How do clients' needs and requests influence your food pantry's fresh food offerings?

66 m 11. Could you describe your relationship with the regional food bank and their role in providing fresh foods to your food pantry?

72 m 12. If there was an increased amount of fresh foods available through food banks, what considerations should be kept in mind for that to be most effective?
   a. **Prompt**: If you don't currently partner with the food bank, can you speak to what you think would be helpful for other pantries?

Ending Question(s). To end, we would like to focus on next steps to change the amount of fresh foods available at food pantries.

78 m 13. If you received a grant of $5000 or more tomorrow, what would be your top priority for spending that money?
   a. **Prompt:** In other words, if you were offered funding to assist with your mission, how would such funds be best used?

84 m 14. Out of all of the things we discussed today, what do you believe is the most important?
   a. Is there anything we should have talked about today but didn't?

General Probes:
How do you think this could/might work at other food pantries?
What do you think contributed to that?
Can you tell me more about that?
Do you have a specific example that you're thinking of?
How has COVID-19 influenced this?

88 m 15. Now I am going to quickly summarize what we discussed today to ensure that I accurately captured our conversation.

#### MODERATOR SUMMARIZES CONVERSATION BASED ON NOTES ####

Does that sound right to you?

CLOSING:
Thank you all for coming and providing such a rich discussion. I am going to finalize the recording now.

Milk

Examples of Fresh Food at Food Pantries

This is a list of foods that are considered fresh options.
When we discuss fresh foods today, these are the types of foods we are referring to.

Dairy

- Unflavored or unsweetened milk
- Cheese
- Yogurt
- Butter

Fruits and Vegetables

- Fresh with nothing added

Eggs

- Whole eggs in the shell

Unprocessed Meat

- Cuts of meat such as:
  - chicken
  - ground beef
  - pork
